# Evolutionary Characters, Phenotypes and Ontologies: Curating Data from the Systematic Biology Literature

**DOI:** 10.1371/journal.pone.0010708

**Published:** 2010-05-20

**Authors:** Wasila M. Dahdul, James P. Balhoff, Jeffrey Engeman, Terry Grande, Eric J. Hilton, Cartik Kothari, Hilmar Lapp, John G. Lundberg, Peter E. Midford, Todd J. Vision, Monte Westerfield, Paula M. Mabee

**Affiliations:** 1 Department of Biology, University of South Dakota, Vermillion, South Dakota, United States of America; 2 National Evolutionary Synthesis Center, Durham, North Carolina, United States of America; 3 Department of Biology, University of North Carolina at Chapel Hill, Chapel Hill, North Carolina, United States of America; 4 Department of Biology, Loyola University Chicago, Chicago, Illinois, United States of America; 5 Department of Fisheries Science, Virginia Institute of Marine Science, College of William and Mary, Gloucester Point, Virginia, United States of America; 6 Academy of Natural Sciences, Philadelphia, Pennsylvania, United States of America; 7 Institute of Neuroscience, University of Oregon, Eugene, Oregon, United States of America; Max Planck Institute for Evolutionary Anthropology, Germany

## Abstract

**Background:**

The wealth of phenotypic descriptions documented in the published articles, monographs, and dissertations of phylogenetic systematics is traditionally reported in a free-text format, and it is therefore largely inaccessible for linkage to biological databases for genetics, development, and phenotypes, and difficult to manage for large-scale integrative work. The Phenoscape project aims to represent these complex and detailed descriptions with rich and formal semantics that are amenable to computation and integration with phenotype data from other fields of biology. This entails reconceptualizing the traditional free-text characters into the computable Entity-Quality (EQ) formalism using ontologies.

**Methodology/Principal Findings:**

We used ontologies and the EQ formalism to curate a collection of 47 phylogenetic studies on ostariophysan fishes (including catfishes, characins, minnows, knifefishes) and their relatives with the goal of integrating these complex phenotype descriptions with information from an existing model organism database (zebrafish, http://zfin.org). We developed a curation workflow for the collection of character, taxonomic and specimen data from these publications. A total of 4,617 phenotypic characters (10,512 states) for 3,449 taxa, primarily species, were curated into EQ formalism (for a total of 12,861 EQ statements) using anatomical and taxonomic terms from teleost-specific ontologies (Teleost Anatomy Ontology and Teleost Taxonomy Ontology) in combination with terms from a quality ontology (Phenotype and Trait Ontology). Standards and guidelines for consistently and accurately representing phenotypes were developed in response to the challenges that were evident from two annotation experiments and from feedback from curators.

**Conclusions/Significance:**

The challenges we encountered and many of the curation standards and methods for improving consistency that we developed are generally applicable to any effort to represent phenotypes using ontologies. This is because an ontological representation of the detailed variations in phenotype, whether between mutant or wildtype, among individual humans, or across the diversity of species, requires a process by which a precise combination of terms from domain ontologies are selected and organized according to logical relations. The efficiencies that we have developed in this process will be useful for any attempt to annotate complex phenotypic descriptions using ontologies. We also discuss some ramifications of EQ representation for the domain of systematics.

## Introduction

Variation in observable features, or phenotypes, is intensely studied and richly documented within and between species in the literature of systematic biology (e.g., [1]_msocom_2), between wild-type and mutant lines in model organism databases (e.g., [2]), and among genetic phenotypes of humans (e.g., [3]). Although fundamentally important to our understanding of genetics, development, and evolutionary relationships, phenotypic descriptions exist almost exclusively in a free-text or natural language format that is not amenable to computational processing. For example, the diverse ways of describing the shape of the first infraorbital bone in fishes (“lacrymal bone … flat” [4]; “lacrimal … triangular” [5]; “first infraorbital (lachrimal) shape…flattened” [6]) _msocom_3might seem obviously similar to a human but would not be recognized as similar by a computer. Natural language, although allowing the expressive and precise description of biological form, has serious limitations for comparing or integrating data across studies, linking to genetic databases, and data mining.

To facilitate comparison and integration of phenotypes across organisms, model organism communities have spearheaded the representation of mutant phenotypes using ontologies and formal semantics [7]. An ontology extends the notion of a controlled vocabulary by associating names with formally defined entities, which include classes and relationships among those classes (c.f., [8]). Here we use ‘term’ to refer to those names associated with classes, in contrast to names of relationships. The application of ontologies to the curation of phenotype data from the model organism literature and sharing of these annotations in community databases has promoted clarity in communication among researchers and allowed for integration of large quantities of data. In addition to facilitating interoperability among databases, ontologies allow users to query using very specific or broad anatomical terms and obtain organized groups of annotations. For example, a query with the term *dorsal fin* will also return *dorsal fin ray* because of its *part_of* relationship to *dorsal fin*. The Entity-Quality (EQ) formalism, which combines ‘entity’ terms from an anatomical or other ontology (e.g., ontologies that describe observable organism features such as behavior), with non-taxon-specific ‘quality’ terms from the Phenotype and Trait Ontology (PATO) [9], [10], [11] has been employed in phenotype descriptions of model organism mutants, where it has been shown to facilitate the identification of biologically similar phenotypes in different species [12]. Ontologies and the EQ formalism have also recently been applied to the standardization of taxonomic descriptions [13]. While the curation of data from the literature, and the annotation or tagging of those data using ontology terms, may be practices that are less familiar to evolutionary biologists than those in molecular genetics communities, they are nonetheless closely analogous to the curation of museum specimens and their associated metadata, such as locality.

**Figure 1:**
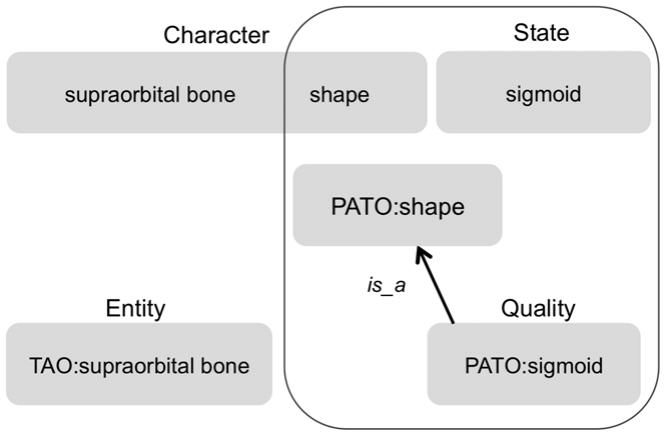
A systematic character and state, compared to a phenotype represented by Entity-Quality syntax. In EQ syntax, the entity being described is represented by a term from an anatomical ontology, and the variable (“characteristic”) aspect of the entity is represented using a term chosen from a quality ontology. Note that the “shape” attribute is an explicit part of the systematic character but is expressed only implicitly within the quality term.

Phenotypic variability across species has been documented in rich natural language in the comparative literature of evolutionary biology and most formally in phylogenetic systematics. This variability is described in systematic characters, which consist of two or more character states contrasting some aspect (e.g., morphology, behavior) of the taxa under study [14]. Character states are assigned to taxa in a character-by-taxon matrix that is analyzed with phylogenetic methods to infer hypotheses of evolutionary relationships. The EQ formalism has been suggested as a means to integrate data across systematic studies and with phenotypes and genetics of model organisms [15], [16], [17]. For example, a character may describe how a structure (e.g., supraorbital bone) and its attribute (e.g., shape) vary among taxa (Figure 1); the character states specifying the value of the attribute (e.g., sigmoid). In comparing EQ syntax to systematic characters, the quality term represents the character state and, by implication through the subtype relationships of the quality ontology, the attribute of the character (e.g., *sigmoid* is a subtype of *shape*, Figure 1).

Software tools specific to the type of data being curated have proven to be a critical ingredient of an efficient annotation workflow (e.g., for journal articles using Textpresso [18]; or gene structures using Apollo [19]). To link ontology terms with phenotypic systematic characters and taxa, we developed the Phenex curation tool [20]. Upon launch, Phenex automatically downloads the most recent versions of the required anatomy, quality, taxonomy, and other ontologies. Phenex allows curators to use EQ syntax to represent evolutionary characters [16], [17]. Using Phenex, this simple combinatorial EQ syntax can be elaborated, for example, to accommodate multiple related entities and to describe complex entities.

As part of the Phenoscape Project (http://www.phenoscape.org), which aims to integrate model organism with evolutionary phenotype data using ontologies, we mounted a large-scale initiative to curate a significant data set of evolutionarily-varying phenotypes from the phylogenetic systematic literature. Specifically, we curated 4,617 characters pertaining to a monophyletic group of teleostean fishes, the Ostariophysi (catfishes, characins, knifefishes, carps, and minnows; [21]), which also includes the model organism, zebrafish (*Danio rerio*). There are many decisions and details involved in the implementation that are not necessarily intuitive or straightforward; these decisions affect how the annotated data can be used. As a result of this experience, we recommend standards and procedures that will enable more consistent and efficient translation of complex phenotype descriptions into computable data. These in turn will enable accurate character and phenotype comparisons and integration on a much broader scale. The principles and best practices for the curation of complex phenotypes that we have developed from this exercise are generally applicable, as are the challenges inherent in aligning rich textual descriptions with ontologies and syntactic relations.

## Methods

### Curation software and ontologies

For annotation of the 47 studies from the fish phylogenetic literature with EQ syntax, we used Phenex [20], the annotation software that we developed for evolutionary biologists to link phenotype descriptions (characters and character states) with ontology terms. Phenex can be configured to load any ontology in OBO (Open Biological and Biomedical Ontologies; [7]) format. We configured it to load the Teleost Anatomy Ontology (TAO) [15] and Teleost Taxonomy Ontology (TTO) (Midford et al., in prep), in addition to several other shared community ontologies including the Phenotype and Trait Ontology (PATO), Gene Ontology (GO), Spatial Ontology (BSPO), Relations Ontology (RO), Evidence Code Ontology (ECO), and Unit Ontology (UO). These ontologies are available for download from the OBO Foundry [22]. A list of museum codes [23] derived from the Catalog of Fishes [24] was also loaded into Phenex. Phenex files were saved in NeXML format, a phylogenetic data exchange standard that permits systematic data to be tagged with ontology terms [25].

### Literature selection and collection

An initial list of 420 studies, including published species descriptions, taxonomic revisions, phylogenetic studies, and unpublished theses and dissertations, was compiled from suggestions by 10 experts on the morphology of ostariophysan fishes and close relatives. These experts helped prioritize the list to emphasize studies on higher-level groups for broad taxonomic coverage, and studies that included data matrices for the efficient annotation of phenotypic characters. We describe here the results of curation of all characters reported in a total of 47 studies on ostariophysans, their clupeomorph relatives, and some euteleosts (percomorphs and salmoniforms). These studies were published between the years 1981–2008. They included 26 peer-reviewed publications [4], [6], [21], [26]–[48], 12 book chapters [49]–[60], eight Ph.D. dissertations [5], [61]–[67], and one M.S. thesis [68]. Because this collection of studies spans the decades prior to the widespread availability of publications in electronic format, we obtained electronic versions from numerous sources: approximately half (23) were scanned from hard copies with text translated by Optical Character Recognition (OCR), 19 were obtained online from institutional libraries, two hard-copies were acquired through Interlibrary Loan, one was purchased from ProQuest UMI Dissertation Publishing, one was downloaded from the Biodiversity Heritage Library, and one was obtained from the corresponding author.

### Curator training, curation experiments, and quality control

Curation of the complex phenotypic descriptions contained in systematics publications required the input of domain experts who were knowledgeable on the anatomy of the target taxa. Curation was done by five ichthyologists under the direction of a lead curator (W. Dahdul). Curators were trained one-on-one by the lead curator at annotation workshops or remotely by conference calls. A Guide to Character Annotation was maintained on the Phenoscape wiki [69] that kept curators up-to-date on the developing best practices for curation. The phenoscape-curators mailing list [70] was used for discussion and communication of data curation issues, solutions, and progress. Participation in and discussion of issues on several OBO Foundry [22] community mailing lists, particularly obo-discuss [71] and obo-phenotype [72], also contributed to the development of standards for the curation process.

As part of our curation quality control, we conducted two annotation experiments at Phenoscape project workshops to identify areas of improvement in curator training, ontology development, and software tools. We wanted to determine how often, and for what reasons, curators choose divergent EQ statements for the same character and character states. Curator training consisted of a hands-on group annotation exercise, and at least one full day of individual work on each curator's own publications with assistance from the lead curator and other project personnel. The Guide to Character Annotation [69], with examples of character types commonly encountered in the fish systematic literature, was also provided to the curators. In both curation experiments, the same 10 characters sampled from the ichthyological literature were annotated by 4 or 5 curators in parallel.

### Curation workflow

**Figure 2:**
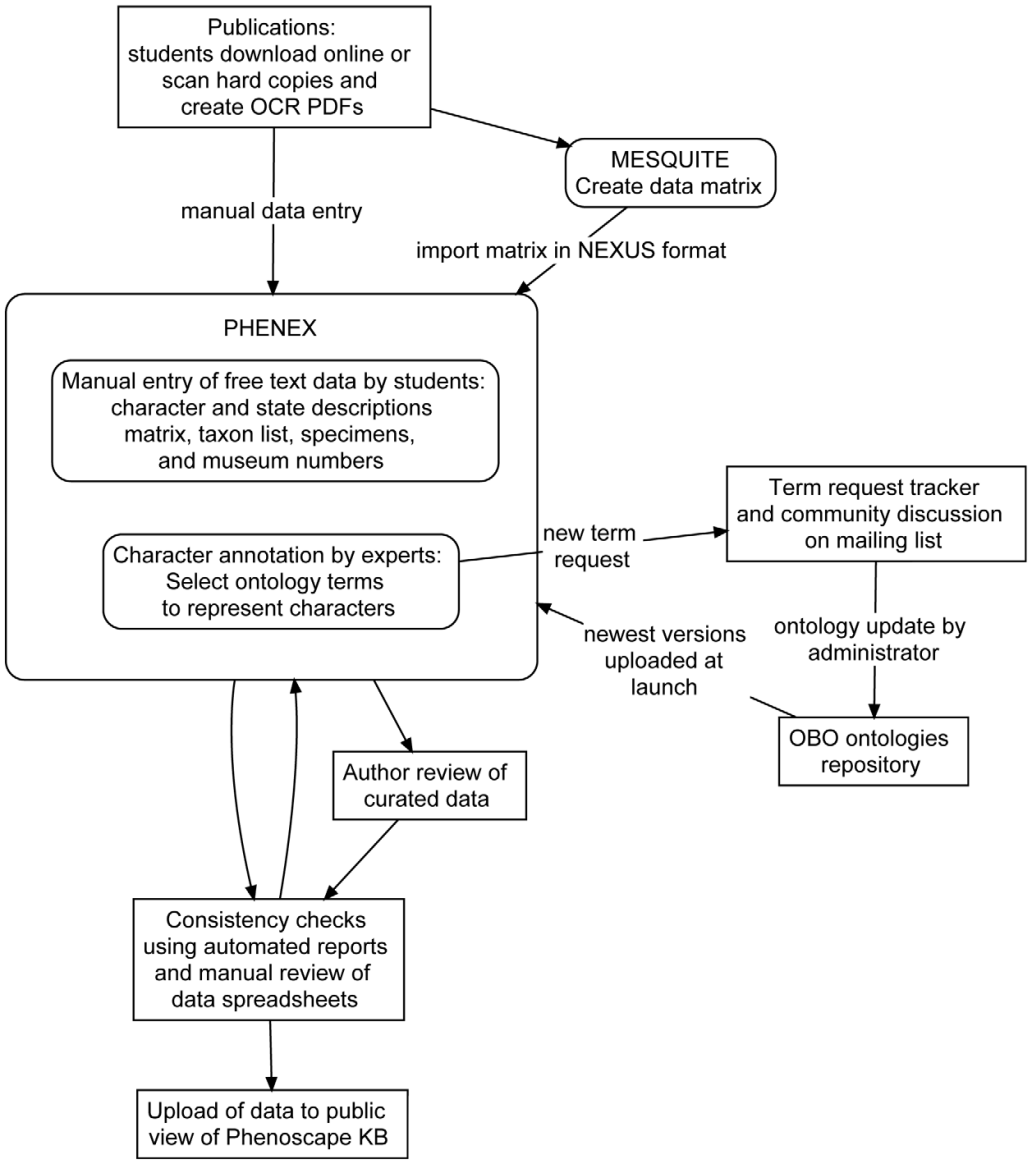
Workflow for the curation of phenotypic characters from systematic studies.

The workflow for curation of publications (Figure 2) required the coordinated activities of students, taxonomic and anatomical experts, the use of specialized curation software (Phenex), online tools, and community input.

#### Free text entry

Free-text data were manually entered into Phenex by undergraduate student workers. The data entered included character and character state descriptions, taxon names, phylogenetic matrices, and specimen collection numbers. Although a few matrices were obtained from authors, most were transcribed by undergraduate students from the original publications using Mesquite [73], and subsequently imported as NEXUS files into Phenex. Matrices for our publications of interest had not been deposited into public data repositories such as TreeBASE (http://treebase.org), an online source of user-contributed phylogenetic matrices.

Materials (species and specimen) lists were also manually recorded using Phenex. Specimen information in the Materials section in the systematic literature is customarily organized by species, and sometimes by a higher-level taxonomic category such as family. Species names are followed by the examined voucher specimens, their institutional catalogue acronyms and numbers, and often by the number of specimens (in parentheses). The number of specimens is frequently qualified by the number in the lot that were examined and/or the number that were prepared differently (e.g. cleared and stained for bone and/or cartilage, radiographed, dry skeleton, muscles, alcohol-preserved). We curated only those specimens that were prepared for the observations that the authors documented in the character statements (typically only skeletal morphology). Additionally, some authors provided the size range of individuals in the collection, and abbreviated locality information, and they sometimes indicated whether the specimen(s) forms part of the species type series; such data were not curated.

#### Selection of ontology terms for taxa

The Materials list and character matrix of each systematics publication contain names of the taxa and individual specimens (voucher specimens) that were examined by the authors. These form the basis of observations for phylogenetic characters. Taxon names from the Materials list and matrix were linked to currently accepted (according to the Catalog of Fishes, CoF, [25]) taxon names from the TTO by undergraduate student workers using Phenex. A taxonomic expert then reviewed the taxon list and, after verifying taxonomic status in the CoF, requested addition of names or synonyms missing from the TTO using the SourceForge term request tracker [74]. Unknown or unidentified species were added to the TTO with reference to the publication in parenthesis (e.g., *Akysis sp. 1 (de Pinna 1996)* TTO:10000093, *Akysis sp. 2 (de Pinna 1996)* TTO:10000094). Most synonyms were added to the TTO with a scope of RELATED (rather than BROAD, NARROW or EXACT), indicating that the relationship between the synonym and its primary term was not known. Species names incorrectly spelled by an author were added to the TTO as synonyms with a scope of EXACT and an associated synonym category of ‘misspelling.’ The eleven misspellings and missing taxon names discovered in the CoF through this process were communicated to the CoF administrators for correction or addition.

Some publications partially or wholly replicated the species names from the Materials list in the phylogenetic matrix. However, many publications used higher-level taxa (e.g., genus, family) for the taxonomic units represented in the matrix. Because we recorded phenotypes as properties of species unless specifically asserted to a higher-level taxon by an author, any higher-level taxon used in a matrix was replaced by all the species within that taxon as listed in the Materials list. This procedure sometimes required contacting a taxon expert for assistance in assigning species to the correct higher-level taxon in the matrix.

#### Selection of anatomy ontology terms for representation of character states

When curators encountered a term in the literature that was not in an ontology, they first assessed its use and context in the publication to determine whether it was a new term or a synonym of an existing term (synonyms include misspellings). This involved reading all uses of the term in the paper and checking figures to see whether the author provided further information. Sometimes this also required searching the referenced literature pertaining to the term. If it was deemed to be a new term, the curator wrote a corresponding genus-differentia definition [7] and proposed the relationships of that term to other terms in the ontology. For example, a term was requested for the hypomaxilla, a bone of the upper jaw in clupeomorph fishes. The request included a proposed definition, “Dermal bone found in the anterior margin of the upper jaw, posterior to the premaxilla,” and proposed relationships to other terms (*is_a dermal bone*, *part_of palatoquadrate arch*, *part_of dermatocranium*). The curator submitted this request through the TAO SourceForge Term Tracker [75], which triggered an automated email to the community mailing list [76]. The ontology administrator closed the request after the conclusion of mailing list discussion, and then updated the ontology to include the requested change and associated community comments. A similar term request procedure was followed for quality terms needed for PATO.

An alternative to adding terms to an ontology is to create a new term at the time of annotation by post-composition, which is the process of combining terms from one or more ontologies to create a new term ([77]; also see Guide to Character Annotation in Text S1). Frequently, curators needed terms for the processes, margins, and regions of specific structures. Rather than adding these directly to an ontology, relevant terms from the spatial ontology (e.g., *anterior margin*) and anatomy ontology (*frontal bone*) can be joined by a relation (*part_of*) to create a post-composed term (the *anterior margin* that is *part_of* the *frontal bone*). Generally, terms were post-composed when they were not expected to be used repeatedly in annotation. Those known to exist in multiple species and referenced repeatedly in the literature were added to the anatomy ontology (e.g., *supraoccipital process*).

#### Granularity of curation

**Figure 3:**
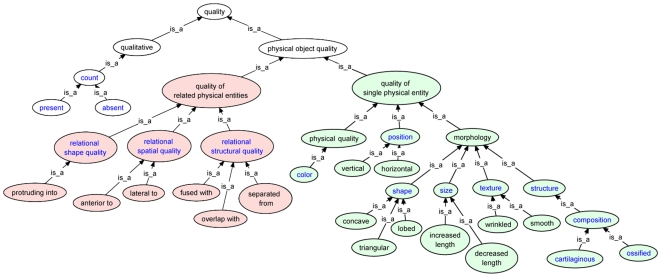
Attribute-level quality terms from the Phenotype and Trait Ontology used to curate systematic characters. Terms in blue font represent the higher-level concepts used to describe phenotypic variation at a coarse level (see Figure 4). Qualities are divided into those that inhere in a single entity (*quality of single physical entities*; green fill) and those that inhere in multiple entities (*quality of related physical entities*; red fill). Examples of children of some terms are also shown.

**Figure 4:**
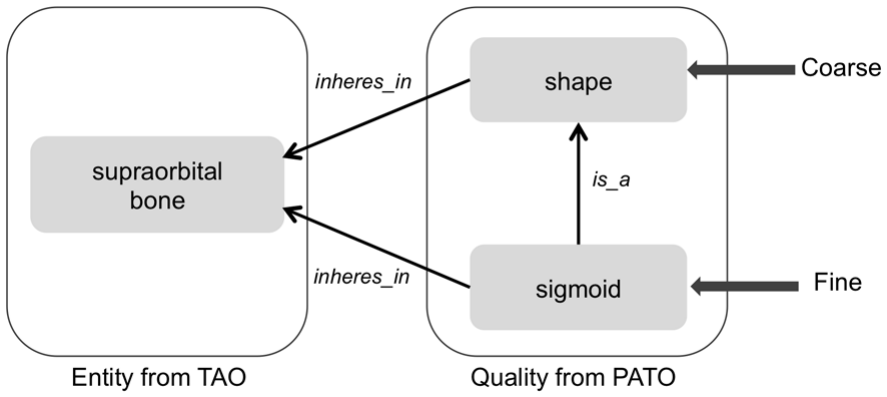
Comparison of coarse-level and fine-level phenotype annotations for the observation of a sigmoid-shaped supraorbital bone.

To maximize curation consistency among curators and to meet the needs of the larger purpose of our work, which is to integrate the phenotypic data of evolutionary morphology with phenotype descriptions of zebrafish mutants, we did a ‘first pass’ curation of the characters to a coarse level of granularity. By coarse, we mean that we selected higher-level terms, or those with less specificity, for quality and sometimes entity. Coarse-level qualities from the Phenotype and Trait Ontology (PATO) are those at the attribute level such as *size*, *shape*, and *composition* (i.e., those terms in blue font in Figure 3). The supraorbital bone, for example, is described as having a sigmoid shape in some characiform fishes [48]. The coarse-level EQ annotation for this phenotype is E:supraorbital bone, Q:shape, whereas the fine-level annotation is E:supraorbital bone, Q:sigmoid (Figure 4). Coarse annotation meets the immediate use of linking to zebrafish genetic phenotypes in the Phenoscape Knowledgebase (http://kb.phenoscape.org), because most of the zebrafish phenotypes are currently annotated to a coarse level by ZFIN. In addition, the coarse-level annotations, though lacking the detail that free-text provides, do express the author's assertion that a change in some aspect of shape is evident between species. Annotations at this coarse level, i.e., *shape*, allow aggregation of all entities and species that have experienced an evolutionary change in shape. After curation of the 47 papers at a coarse level was complete, we did a ‘second pass’ of finer-scale curation of qualities by selecting a more specific child term and finer-scale curation of some entities by using post-composition.

#### Evidence codes for phenotype observations

We recorded phenotype descriptions as properties of species, and these were assigned one of three evidence codes based on the type of evidence given by an author. These codes are part of the Evidence Codes Ontology [78], which is used by the broader biological community. A phenotype description that is explicitly tied to a specimen was assigned IVS (Inferred from Voucher Specimen); these referenced an institutional catalog number. A phenotype description in which the author does not reference a specimen was given one of two weaker evidence codes (NAS, Non-traceable Author Statement, or TAS, Traceable Author Statement); no catalog number could be associated. NAS is used for statements that an author makes with no results or citation presented. TAS is used for author statements that are attributable to another source. This same methodology was extended to statements about higher-level taxa. Here, species-level phenotype annotations were generated for every species included in the higher-level taxon (as listed in the Materials list). In this case, these particular species were given a strong evidence code (such as IVS) with catalog numbers attached. When the author did not reference the species that were observed to make character assertions about higher-level taxa, the higher-level taxa were assigned a weaker evidence code. The phenotypes described in most of the publications that we curated were based on observations of voucher specimens and so merited the strong IVS evidence code.

#### Review of annotations for consistency among curators

Annotation summary reports in the form of a spreadsheet containing annotations for all 47 publications were generated regularly from the Phenoscape Knowledgebase to review annotation consistency. Additionally, Phenex can export files in Excel format, so that consistency can be checked for individual files. We developed a series of review tasks that checked for proper EQ syntax and consistent annotation of different character types. These include checking that a related entity was recorded when a relational quality was used; checking that a related entity was not recorded with a quality of a single entity; checking for incomplete annotations (e.g., only one state annotated); and checking that post-composed terms were nested correctly (e.g., *process* (*part_of* (*anterior region* (*part_of* (*maxilla*)))) versus *process* (*part_of* (*anterior region*))((*part_of* (*maxilla*))) and created with logically correct relations (e.g., *part_of*, *connected_to*, *overlaps_with*, *adjacent_to* and spatial relations such as *anterior_to*). Undergraduate student workers also proofread the curated files to check for the correct transcription of data matrices and numerical values of counts.

#### Author contact and verification

fter the curation of each publication was completed and verified for consistency, the primary author was notified by email that their published data had been curated for inclusion in the Phenoscape Knowledgebase. Authors were sent a spreadsheet with the original character and taxonomic data and its ontological representation (e.g., free-text character descriptions vs. EQ phenotypes; published vs. currently accepted taxonomic names). Authors were invited to send suggestions or corrections prior to upload of the data to the public version of the Phenoscape Knowledgebase. Two authors returned corrections to their published data.

#### Data upload to Phenoscape Knowledgebase

Phenotypes and corresponding matrix information were loaded into the Phenoscape Knowledgebase, a relational database built on the Ontology-Based Database (OBD) schema, in which all data are represented as semantic links between ontology terms (Kothari et al., in preparation). The deductive reasoning and the query interface of OBD support analyses of the anatomical and taxonomic disposition of phenotypic annotations at any level of granularity that is present within the logical structure of the ontologies. In addition to the Knowledgebase itself, we created a web interface (http://kb.phenoscape.org/) that allows users to browse and query the phenotype data in ways that exploit the ontological context.

## Results

### Characteristics of the data reported in the source publications

From our comprehensive undertaking to represent complex phenotype data using ontologies, patterns emerged in how characters, taxa, specimens, and matrices were presented in the different studies. These led to the creation of annotation guidelines (summarized in Text S1; [69]). The standards and the variation encountered in the literature also drove the development of our annotation software (Phenex).

#### Character and character states and EQ annotation

The curation of 4,617 characters, or 10,512 character states, resulted in 12,861 ontology-based phenotypes or Entity-Quality statements. Characters and character states were divided among several categories in the process of EQ annotation. First we distinguished among characters and states that involved a single entity vs. those that involved two or more entities, terminology that follows the division of quality terms in PATO [11]. For example, a character might involve a single anatomical structure, such as the shape of the dorsal fin (“dorsal fin … acuminate” [49]) versus a character that involves the relationship between two structures (“dorsal fin origin anterior to that of pelvic fin” [49]). Selection of specific entities from the appropriate ontology was generally the next step in the curation process (described further in Discussion). The third step was to determine the particular quality, initially at the attribute level, that is required to represent the phenotype described in the character state. If a single entity is involved, a monadic quality (*quality of a single physical entity*; Figure 3), i.e., one that inheres in a single entity is required. If two (or more) entities are involved in the phenotype, frequently a relational quality (*quality of related physical entities*; Figure 3), i.e., one that inheres between multiple entities, is required. Size comparisons among entities require special consideration, and involve monadic qualities (see Text S1). Last, we considered whether a character state contained single or multiple logical qualities and thus required single (non-composite characters) or multiple (composite characters) EQ statements. Frequently we found that several different attribute qualities (e.g., *color* and *shape*; Figure 3) were required for the annotation of composite characters (see Systematic Character Types and Application of EQ Formalism).

#### Taxonomic names

From the 47 publications, we curated phenotype data to 3,449 taxa (mostly species), of which 2,682 were nonredundant names, of which there were a corresponding 2,410 valid names (according to the Catalog of Fishes, CoF [24]). Of the 2,682 names, 729 are now invalid or were misspelled. The invalid names were annotated to the currently valid name as listed in CoF using the TTO, or the invalid or misspelled name was added as a synonym, if not already present in CoF. Three hundred and ten taxon names were added to the TTO as unknown, uncertainly identified, or unnamed taxa (out of 36,895 taxonomic terms total). We included reference to the author(s) and year in the term name for these 310 publication-specific taxa (e.g., *Akysis sp. 1 (de Pinna 1996)*, TTO:10000093).

#### Specimens and materials examined

In most of the publications (42 of the 47) a list of materials was presented, giving the provenance and other information about the particular specimens that were examined. Synthesis papers [55] or book chapters [51], [52] frequently did not include a Materials list, but specimen collection numbers were sometimes provided in figure captions (e.g., [36]). We obtained a list of materials examined from the authors of these papers where possible. Some authors referred to previous publications for a full list of materials (e.g., [28], [59]), and where feasible, we curated specimens from these.

#### Character-by-taxon matrices

Forty-five of the 47 curated publications included data matrices. From the two publications that lacked them, one was supplied by the author [4] and the other was reconstructed from the text [21]. Some matrices contained numerical character states that were not textually described. We annotated EQ statements for only those character states that were documented.

Higher-level taxa appeared in 35 of the 45 published data matrices. These taxa were expanded to the species level to represent the particular species examined in a publication. As mentioned previously, this was not always straightforward because some authors did not indicate which species belong to these higher-level taxa used in the matrix. For example, one author [63] categorized species by family in the Materials list, but additionally used subfamilies in the matrix. In this case, curation of species to the correct matrix subfamily (and thus to the correct phenotype descriptions) required personal communication with an expert taxonomist. Additionally, some authors (e.g., [65]) organized species in the Materials list by higher-level taxa proposed in their own study (and reflected in their matrices) instead of by currently recognized higher-level taxa. Again, curation of species to the correct matrix taxon, and thus to the correct character data, required personal communication with an expert taxonomist.

### Ontology growth

As a result of literature curation, the ontologies that we used grew in number of new terms, synonyms, definitions, and relationships among terms. The TAO more than doubled its skeletal terms, from 253 in version 1 [15] to 644 skeletal terms of 2,662 total in the most recent version (March 2010). The TTO grew to 36,895 taxon terms, including 154 fossil taxa (from an initial 36,080 terms with no fossil taxa), 43,215 synonyms (from an initial 38,269), 30,865 species, 5,107 genera, and 551 families. Our curators contributed 16 terms, four synonyms, three name changes, and one relationship change to PATO. We added four terms (*parental care*, *oral incubation*, *adult foraging behavior*, *foraging by probing substrate*) to the Biological Process hierarchy of the Gene Ontology (GO-BP), and 41 terms and six synonyms to the Spatial Ontology. Museum codes were based on the CoF list [79] (479 entries) that was enhanced with 37 additional codes identified during the process of paper curation.

### Curation experiments and curator consistency

In the first curation experiment, only one of 10 characters was annotated identically among four curators. The reasons for this variability among curators included curation software bugs, difficult aspects of the ontologies (e.g., lack of appropriate quality terms from PATO), lack of standardized guidelines for unusual cases, and differing interpretations of the text descriptions. For the second experiment, curators were told to curate characters to a coarse level of granularity for quality. In this experiment, a greater proportion of characters were annotated correctly to the higher-level quality term (Figure 3) although only two of the 10 characters were annotated identically among curators for the more specific child term. The overall variability in annotation consistency resulted from different interpretations of shape and size descriptors, inexperience and unfamiliarity with the ontologies and software, difficulties in creating post-compositional terms, and lack of adequate terms in the ontologies, particularly for shape descriptors.

### Curation effort

The time required for curation of the chosen papers [4]–[6], [21], [26]–[68] to the level described herein was approximately 5 person-years. This included significant time investment by personnel in software development, testing and improvement, initiation of new ontologies, and development of curation standards and workflow.

## Discussion

Our experience in successfully transforming a large collection (10,512 character states) of legacy systematic character data into the ontology-based EQ syntax resulted in a recognition of several distinct systematic character types with respect to the logical categorization enforced by ontologies. It also contributed to the growth and improvement of several domain and community ontologies, and it resulted in the development of standards and best practices for phenotype curation. Moreover it offers a new view of morphological characters that is valuable for practicing systematists.

### Systematic character types and application of EQ formalism

Systematists use the expressiveness and richness of natural language to describe precisely the morphological variation that they observe among species. These phenotype descriptions are represented in a somewhat formalized way as characters and character states in the systematic literature [14]. EQ formalism provides a rigorous yet flexible syntax for these data; it is to some extent, a ‘natural fit’. As previously described (Figure 1), systematic characters typically consist of a short character header (e.g., maxilla shape) denoting the relevant structure(s) (entity: maxilla) and attribute (high-level quality: shape) that varies among taxa, followed by several character states that specify the value of the quality (round, rectangular, triangular, etc…). Many systematic characters, however, do not follow this format (see [14] for review), and a standardized logical framework, consistent with EQ formalism, has been recently proposed [14]. In systematic characters, entities and qualities can be found in the character description, in the character state description, or in both. Irrespective of this, the morphological descriptions of variants among species in the literature conform to the general formalism of EQ syntax and semantics. From the breadth of our curation work emerged standards and recommendations for deploying this formalism, and we relate these below and in our Guide to Character Annotation ([69]; Text S1).

Systematists typically represent only one aspect of a structure in a character state. An example from bird systematics involves variation in the shape of the external naris in stem rollers: “ovoid (0); triangular with a flat ventral margin (1)” [80]. Here each character state corresponds to a single phenotype: state 0, for example, corresponds to the EQ: E:external naris, Q:ovoid. Occasionally, however, authors represent observations in ways that may be interpreted as either monadic or relational. An example from fishes [48] is a character involving two elements of the anal fin described as: “Presence or absence of fusion of medial and proximal anal-fin radials: (0) absent; (1) present.” Rather than annotate this as a monadic character by adding a new term to the anatomy ontology, i.e. “fused medial and proximal anal-fin radials”, which is a complex entity not named in the literature, we annotated this as a relational character using the qualities *fused with* and *separated from* to describe the relationship between two separate entities (state 0: E:medial anal-fin radial, Q:fused with, RE:proximal anal-fin radial; state 1: E:medial anal-fin radial, Q:separated from, RE:proximal anal-fin radial). The advantage of representing this using two separate recognized and defined entities is that they can thereby be linked to other annotations of these entities.

Character states were generally translated into a single EQ statement, but not uncommonly we noted that multiple aspects of a structure or multiple structures are described within a single character state, requiring the annotation of multiple EQ statements. This may reflect an investigator's observation that the structures co-vary, and perhaps an assumption that they are non-independent and thus represent a single character state. We termed these ‘composite’ character states. For example, variation in the pectoral fin is described as follows [26]: “Pectoral fin size. 0: pectoral fin large and pigmented; more than 43% head length; membrane infused with numerous small chromatophores; 1: pectoral fin small and unpigmented; less than 43% head length; membrane without chromatophores.” Here each character state corresponds to two phenotypes (e.g., state 0, EQ1: E:pectoral fin, Q:size^∧^increased_in_magnitude_relative_to (E:pectoral fin in_taxon X) and EQ2: E:pectoral fin, Q:pigmented). Dividing the distinct logical components (size and color in this case) into multiple EQ statements is necessary for reasoning with them independently using ontologies – and possibly, but not necessarily, for phylogenetic character construction. By recording separate EQ statements, one may query on independent logical qualities of a character state (e.g., *size*) and expect to find similar annotations. However, if a systematist separates them into separate characters, it results in increasing the weight of potentially non-independent characters in the phylogenetic analysis. On the other hand, representing them as a single character may underrepresent them in the analysis.

Systematic characters are sometimes framed such that different character states involve different logical qualities (e.g., *absent* and *shape*). For example, variation in the spermatophoral gland in brachiopods (a phylum of invertebrates) is described with three states [81]: “Absent, Simple, Composite”. Although some systematists have previously raised concerns about the logical structure of these characters, it does not pose a problem for phenotype annotation from a practical standpoint, e.g., the EQ statements for these character states are: E:spermatophoral gland, Q:absent; E: spermatophoral gland, Q:simple; E: spermatophoral gland, Q:composite (see Text S1 for discussion of the semantics for *absent* and *present*). From the standpoint of reasoning with ontologies within a database, presence can be implied by an annotation to any quality term (e.g., *tubular* or *lamellar*) other than *absent*.

The natural language used in the original description of systematic characters sometimes corresponds to a term in an ontology that is different from the author's intent; in other words, there is a mismatch between an author's free-text description and the literal match to an ontology term. For example, authors sometimes describe variability in the shape of a structure using terms that are not types of *shape* in the quality ontology. Variation in the pelvic bone, for example, is described as [29]: “Shape of ischiac process: small or posteriorly elongate (0); falciform (1); falciform and strongly developed (2).” To represent “small or posteriorly elongate” using PATO qualities, the qualities *size∧decreased_in_magnitude_relative_to*(E in_taxon X) and *elongated* are applied. However, the parent of these two terms is *size*, not *shape*. A consequence of this mismatch between natural language and the ontology is that querying the resulting annotations for “pelvic bone” and “shape” will not return annotations corresponding to the author's state 0. Thus applying mutually exclusive terminology to annotate these aspects from a morphological description can be difficult and possibly result in misrepresentation of an author's intention. Many investigators, however, would recognize that many aspects of size variation also relate to shape and *vice versa*. Size and shape terms were a frequent source of inconsistency among curators. In cases such as the example above, we recommended that curators annotate coarsely to *shape*.

The process of dissociating the states of some characters (composite) into multiple, distinct EQ statements, and the states of other characters into EQ statements with different logical values has implications for their potential use in phylogenetic analysis. Characters are the units of homology in phylogenetic analysis, and the alternative character states have been judged by the researcher to be homologues of one another. To the extent that they are atomized using EQ, they may lose their genealogical connection. On the other hand, the task of homologizing complex phenotypic characters across different studies and taxa has proved difficult to impossible for phylogeneticists thus far, and it may be the case that EQ statements provide the broad initial grouping of characters and character states that facilitate subsequent broader-scope phylogenetic evaluation.

### Curation of taxa

Our finding that more than one quarter (729 of 2,682) of the species names used in the 47 curated publications were outdated was unexpected, given the recency of these publications (1981–2008). These fishes may present an unusual case, however, because two of the groups that we curated (catfishes and cypriniforms) have undergone extensive recent taxonomic revision. Given that some taxa will be revised more frequently than others, the rapid turnover in taxonomic names draws attention to the need for adaptable resources such as taxonomy ontologies like the TTO, that record the relationships among not only current, but also synonymous, taxonomic names thus supporting comparisons across studies in the literature. Ontologies provide the capability to accommodate the needs of the specific literature or type of data under curation.

Phenotypes are recorded from observations on individual organisms in both model organism genetics and evolutionary biology. Curation of the evolutionary literature, however, presented a special challenge because it required distinguishing between author statements that were based on direct observation of specimens and generalizations to higher-level taxa. We discovered that authors represented species observations using one or more higher-level taxa in more than 75% of the published data matrices that we curated. Generalizing to a higher-level taxon from observations on a single or only a few exemplar species is in fact common practice in systematic studies of many taxonomic groups. Sometimes an author explicitly asserts in the corresponding text that a particular phenotype pertains to all species included in a higher level taxon, but other times using a higher-level taxon in a matrix is simply shorthand for reference to the species that were actually examined. To compare data across multiple studies, however, it is critical to interpret appropriately the meaning of the author's use of these higher-level taxa.

### Curation of phenotypes in the legacy literature: challenges and feasibility

The rich literature that documents the similarities and differences among taxa goes back several centuries, spans many languages, and at first appearance, seems almost insurmountably large to render computable using ontology-based curation methods. By initially focusing on large-scale treatments where phenotypic descriptions are most formalized, i.e., the phylogenetic studies, we reduced the number of papers for more than 8,000 species of ostariophysan fishes to approximately 50. This struck us as surprisingly few such papers; however, given that the phylogenetic approach has been mainstream only over the past 30+ years, and that morphological treatments of this sort may take an author 6–10 years to produce, the number may well be representative for other taxonomic groups. If this is the case, with annotation software and ontologies in place at the outset, we estimate that similar phenotype annotation projects can be done in possibly half the time (approximately 2.5 person years). Additionally, by incorporating semi-automated methods to extract character states from the literature and associate ontology terms, the time involved could be further reduced. A significant level of phenotypic data, however, remains in non-phylogenetic studies, e.g., species descriptions, and methods for efficient EQ curation of this literature remain a critical challenge.

New attempts to curate phenotypes from the legacy literature of other taxonomic groups (phylogenetic treatments or not) will require overcoming the initial hurdle of creating new ontologies or expanding existing ones for the inclusion of terms required to represent the diversity of organism features and taxonomy under consideration. In our experience, we found it efficient to build from existing ontologies where available. We used, for example, the existing PATO ontology for annotation of qualities, and we built the Teleost Anatomy Ontology (TAO) from the Zebrafish Anatomical Ontology [15]. As we annotated the literature, we concurrently added required terms and relationships to these and other ontologies. In this way, ontology growth and development was driven by active curation of the literature. For example, in the course of curating evolutionary phenotypes for the fishes in this study, we more than doubled the number of primary terms (not including synonyms) for the skeletal system axis of the TAO. In contrast, no new terms were proposed for cell types, embryonic structures, or the immune system, because no evolutionary variants of these anatomical structures were documented in the literature we curated. As a consequence, ontologies might appear to be incomplete or missing basic terms, and may not provide the encyclopedic knowledge that some may expect of an anatomy ontology. Although terms, definitions, and relationships can be supplied at any time, the effort required for curators to break away from direct annotation and turn to ontology development is significant; 15–50% of an individual curator's time might be spent on ontology development. In particular, curation of publications covering taxa that have not been previously annotated require the addition of new taxonomy and anatomy terms and is thus more time consuming. We anticipate that future curation of the fish literature will be more time efficient because of our significant refinement and enlargement of the core ontologies (TAO, TTO, PATO). For new efforts in different taxonomic domains, once the respective ontologies have been populated, the curation of additional publications will require less time. In summary, term addition to shared ontologies broadened their scope and provided greater utility to others in the community.

A significant general challenge for newly established curation projects is the consistent annotation of phenotypes among curators. In our experience, curator consistency is influenced by familiarity with the tools, ontologies, and syntax for creation of phenotypes. Consistency improved as curators gained familiarity with the ontologies, acquired experience using curation software and tools, became more aware of the developing annotation standards, and were restricted in their term choices. The almost daily updated documentation of annotation problems, examples, and standards in the Guide to Character Annotation [69] was important in promoting these annotation standards. High-level oversight of the process and manual and automated consistency checks by the lead curator before making the data public were critical to maintaining consistency and data quality, and contributed to improvements in consistency.

The results of our curation experiments and subsequent work with individual curators pinpointed several general problems that required improvement in curation procedures and software tools to increase curator consistency and efficiency. Importantly, we discovered that curators had a difficult time navigating large ontologies to determine whether an appropriate term was present. This is almost certainly a general problem in annotation of phenotype descriptions and not specific to systematic biology literature. The absence of the correct ontology term led to inconsistent use of existing terms by curators or a time-consuming change of focus to the process of term addition and definition. The solutions that we suggest and have at least partially implemented are generally useful for other phenotype efforts, and they are described below.

To help remediate the navigation of large ontologies problem, we implemented software restrictions to reduce the number of terms available for use in annotations. For example, if only the skeleton is being annotated, then a ‘slim’ version of the TAO containing only terms from the skeletal system might be made available. Restricting which terms are available is particularly important in large community ontologies (e.g., the Gene Ontology) that contain many terms not applicable to the particular data under curation. We, for example, implemented a ‘slim’ version of the Relations Ontology, with only a small subset of relations (such as *part_of, towards*, etc.) available for use by our curators. We found that it is critical for relations to be restricted for use in post-composition. Curators, for example, improperly used *left_of* rather than *in_left_side_of* and *contained_in* rather than *located_in*. In the future we feel it will be useful to further restrict availability of particular terms and relations, depending on the literature under curation, so that for example, the relation *connected_to* is the only one available for use when annotating the relation of scales to the body of fishes. Thus a curator would be forced to annotate *scale connected_to head* versus (incorrectly) *scale part_of head*. Such restrictions are expected to decrease the effort required by a curator to find an appropriate term or relation, and increase significantly the consistency of curation and thus the logical value of the annotations.

The second part of the curation problem is that after a curator determined that a term was truly missing from an ontology (versus in some part of the ontology that they may not have browsed), they then needed to request that a new term be added to the ontology. This was a significant interruption in the curation process, as curators turned their attention from the phenotype description to composing a new term definition. To help expedite the term addition process for curators, we provided easy links to term trackers on the Phenoscape wiki [69] for different ontologies. We also encouraged curators to provide basic definitions that could later be improved upon by community feedback on the term request mailing list [15].

Missing terms also led to curatorial inconsistency. The curation experiments showed that curators frequently could not find the appropriate fine-scale quality term in PATO, mainly due to the incomplete development of this ontology (i.e., the term was missing). This led to individual curators choosing different, fine-scale terms that approximated the author's intent, but did not fully or adequately represent it. Rather than expedite the term addition process here, we used the Phenex software to restrict term choices primarily to higher-level attribute qualities (Figure 3). Consistency and efficiency improved as a result. Additionally, term restriction had an educational and training value in that our curators learned to abstract quickly the essence of the varying quality from complex descriptions. The negatives of this approach are that the PATO ontology did not grow at the leaf-node level, at least initially, as a result of our work, and that queries across qualities cannot be made at a fine scale. That is, querying for all ‘elongate’ jaws, for example, would return jaws of all shapes because all descriptions of jaw shape variation are annotated to the high level term *shape*. The cost of further annotation refinement must be judged against the intended use of the annotations.

It is a significant remaining challenge to curate complex phenotype descriptions using EQ syntax fully. This challenge extends beyond systematic biology to all curation efforts (e.g., Human Phenotype Ontology; http://www.human-phenotype-ontology.org) that seek fine-scale representation of phenotypes, including those that compare human genetic phenotypes to those of model organisms. This is because full or fine-scale curation of complex phenotype descriptions (e.g., the antero-dorsally projecting process of the posterior maxilla is located posterior to the laterally projecting and bent knob of the ethmoid in species X) requires elaborate post-compositional combinations of ordered sets of terms from multiple ontologies. All efforts to represent complex phenotypes will require multiple ontologies and post-composition, and they will thus experience the same general problems. Although the Phenex software supports such compositions [20], and although sophisticated and biologically relevant reasoning across these compositions is feasible [77], the curatorial burden of accurate and consistent annotation at this scale is high. Our work to reduce the burden of curation of these phenotypes by developing standards, restricting ontology terms available for annotation, and making it easier to add new terms, represents a significant step forward.

### Conclusions

The benefits of using an ontology in communication in any discipline include standardization of terminology, explicit definitions of concepts, logical relations among concepts, and the creation of structured and precise representations of information that facilitate computability. From a practical standpoint, communities benefit because communication is clearer and less ambiguous. Using multiple ontologies to describe more complex concepts such as phenotypes can promote similar benefits at a broader level and to a broader community, promote comparisons of phenotypes across studies and taxonomic groups, and allow interoperability with different data types. Currently the multiple ways that investigators describe their observations makes it difficult to combine or compare data across studies, and renders the observations vulnerable to misinterpretation. Many of the issues we encountered in curation (different terminologies, noncomparable attributes among character states) could be avoided prospectively if systematists are provided access to data collection tools that link to community anatomy, quality, and taxonomic ontologies. Use of ontologies for complex phenotype descriptions has the potential to clarify the identity of structures under consideration, allow comparison of similar phenotypes, and facilitate the application of characters across studies and taxonomic groups. Moreover, use of a mapping to EQ syntax during the course of a study can generally promote higher levels of standardization.

Curated data that a computer can understand and reason with facilitates the aggregation and comparison of data on a scale that is unmanageable for individual researchers. The expressiveness, creativity, and precise descriptions possible with natural language, however, are not easily replaced, despite the promise and advantages of computational methods. The inherent human ability to describe and interpret complex phenotypes will always be an essential element in biological fields that involve comparisons of the visible phenotype. These, however, are complemented by computational tools such as ontologies that promote clarity and communication among researchers and interoperability of data.

## Supporting Information

Text S1 Phenoscape Guide to Character Annotation. We describe our standard practices for annotation of entities and qualities in the Guide to Character Annotation. Our online version describes more specialized cases and issues (https://www.phenoscape.org/wiki/Guide_to_Character_Annotation).
(0.09 MB DOC)
