# Supplementary material for: Evolutionary Characters, Phenotypes and Ontologies: Curating Data from the Systematic Biology Literature
Source: PLoS One. 2010 May 20;5(5):e10708. doi: 10.1371/journal.pone.0010708 (PMC2873956; doi:10.1371/journal.pone.0010708)
Supplement: Text S1 — Phenoscape Guide to Character Annotation. We describe our standard practices for annotation of entities and qualities in the Guide to Character Annotation. Our online version describes more specialized cases and issues (https://www.phenoscape.org/wiki/Guide_to_Character_Annotation). (0.09 MB DOC) [file pone.0010708.s001.doc]

**Text S1**

Guide to Character Annotation

**I. Ontological representation of entities**

Systematic characters describe phenotypic variation in entities, the features that vary in some quality among taxa. Here we describe our standard practices for annotation of entities. Our online Guide to Character Annotation [1] describes more specialized cases and issues. An entity may be represented in Entity-Quality (EQ) syntax either by an existing term in an ontology or by concatenating ontology terms in the form of a post-composition to create a new term at the time of annotation.

*Choosing the appropriate entity.* Most term selections are straightforward and involve a curator choosing the appropriate term from the relevant ontology. In some cases, however, the inconsistent nomenclature used in the literature, particularly for serially homologous structures, can cause confusion in the process of choosing a term. For example, a character describes variation in a particular rib as: *"*Presence or absence of medial process on central portion of body of first rib" in characiform fishes [2]. The authors refer to the first rib located posterior to the Weberian apparatus as "rib 1", and this rib is associated with vertebra 5 in characiforms. Other authors, however, refer to this rib as the fifth rib. To clarify the identity of rib terms in TAO, ribs are named for the vertebra they are associated with (e.g., *rib of vertebra 5*, TAO:0001751).

*Creation of post-composed entities.* Post-composition (see Methods) is ideal for describing complex entities that may not exist outside of a few species, and thus are unlikely to be required for repeated annotation. Post-compositions can be a source of curator inconsistency regarding the order of term nesting, relationships chosen to link genus and differentia terms, and the level of granularity.

For anatomical structures, post-compositions are often created to represent the processes, regions, and margins of structures. For example, a particular structure, such as the prosoma or cephalothorax in spiders (arachnids), may be represented in an anatomy ontology (e.g., Spider Ontology; [http://www.obofoundry.org](http://www.obofoundry.org/)), but a more specific structure, such as the 'protuberance on the posterior part of the prosoma' [3] may not be. This may not be a term we want to add, either because it is a unique feature of a few taxa and/or because it leads to an unnecessary increase in size of the ontology, causing difficulties for user navigation and software tools. The concepts of ‘protuberance’, 'region', and 'margin' and their locations relative to the body of the structure (anterior, lateral, etc.), can be drawn from the appropriate ontologies and connected using relations, also drawn from an ontology. These post-composed terms are generated using the genus-differentia principle, where one term serves as the genus, which is then differentiated from other sibling terms using a relationship and a differentia term. As a rule, the more general structure is chosen as the genus, and a relationship and differentia term chosen to narrow down the post-composed term. For example, in composing ‘protuberance on the posterior part of the prosoma',  *protuberance* is the genus, and *part_of prosoma* is the differentia. The differentia can be broken down into a relation (*part_of*) and a target or filler term (*prosoma*). Being part of the prosoma is the differentia that distinguishes this kind of protuberance from all other kinds: *protuberance part_of prosoma*. The order in which the terms are composed is important; we can describe *protuberance part_of prosoma* but not *prosoma* *part_of protuberance*.   Often spatial information is specified in a post-composed term and the Spatial Ontology (BSPO) is used in this case. For instance, the above entity is represented as a nested post-composition: *protuberance^part_of(posterior region^part_of(prosoma))*. The order of this nested post-composition is important (e.g., *protuberance^part_of(prosoma)^part_of(posterior region)* is incorrect). Nested post-compositions are different from post-compositions with multiple differentia. Generally, nested post-compositions are used with transitive relations and post-compositions with multiple differentia are used with non-transitive relations.

Post-compositions can be made using a variety of relations. For example, post-composed terms for the contralateral halves of bilaterally paired structures are created using the *in_right_side_of and in_left_side_of* relations, where the perspective is from the standpoint of the organism. For example, the fusion of the paired frontal bones is described in a species of cetopsid catfish [4]. This phenotype is annotated as: E: frontal^in_right_side_of(body), Q: fused with, RE: frontal^in_left_side_of(body).

**II. Ontological representation of qualities**

Qualities are the logical aspects such as shape and size that inhere in a bearer entity. Qualities that are used for phenotype annotation are represented along several major axes of the PATO quality ontology (Figure 3); one branch contains such quantitative qualities as *count*, *present* and *absent*, and the other branch includes qualities that apply to physical objects, i.e., monadic qualities (children of *quality of single physical entity*) and relational qualities (children of *quality of related physical entities*) (Figure 3). Monadic qualities are those that exist in a single entity, whereas relational qualities are those that exist between multiple entities. We describe how we employed these qualities in reference to systematic characters. Our online Guide to Character Annotation [1] describes more specialized cases and issues.

***Count, present and absent***
Variation within a clade often appears in the form of an anatomical entity that is present in some taxa and lacking in others. These characters are annotated directly with either an entity from an anatomy ontology or a post-composed entity and the qualities *present* and *absent.* These qualities are children of *count* in the PATO hierarchy. For example, the presence or absence of teeth on ceratobranchial 5 bone among ostariophysan fishes [5] is annotated with the following two phenotypes: E: ceratobranchial 5 tooth, Q: absent and E: ceratobranchial 5 tooth, Q: present. The use of *present* and *absent* are curatorial shortcuts that reflect common usage in language familiar to curators, and we used these terms to improve the consistent annotation of presence/absence characters. Their use in EQ syntax, however, is illogical because, for example, *absent* refers to the existence of an entity that is asserted to not exist [6]. Therefore, these annotations are later changed to the logically preferable representation using the quality *lacks all parts of type* [6]: E: whole organism, Q: lacks all parts of type, E2: ceratobranchial 5 tooth.

Characters involving counts of entities were annotated using the *count* quality and their literal value. For example, variation in vertebral counts among species of *Danio* [7] is reported as: "total vertebrae count between 31 and 33 (0); 34–38 (1); greater than 38 (3)." This was represented by three phenotypes as follows: EQ1: E: vertebra, Q: count, Count: 31-33; EQ2: E: vertebra, Q: count, Count: 34-38; EQ3: E: vertebra, Q: count, Count: >38.

***Qualities for single and multiple entities***

Qualities for characters that involve single or multiple entities include *shape*, *size*, and *structure* (Figure 3). For example, the presence of “U-shaped alimentary canal” [8,9] is annotated as E:alimentary canal, Q: U-shaped. Some characters describing an aspect of one entity in relation to another entity are annotated using qualities such as *fused with* or *separated from* whereas others are annotated using a post-composition of a quality, such as *size*.

Size is often referred to comparatively in the systematics literature and is properly annotated using a post-composition of the *size* quality. Authors either compare a structure’s size to that of a different structure in the same species or to that of the same structure in different species. In either case, the post-composition of quality uses the relations *increased_in_magnitude_relative_to* or *decreased_in_magnitude_relative_to* to express the comparison to the second entity. For example, size of two different bones within species was described as the first epibranchial bone larger than the second epibranchial bone [10]. This would be annotated as: E: epibranchial 1 bone, Q: size^increased_in_magnitude_relative_to(epibranchial 2 bone). Similarly, when the size of a structure is compared between species, the following convention is used. For example, the fourth epibranchial bone is broad (in twenty-five species of fish in the genus *Cyprinella*) [11]. In this case, where the comparison is between species, it would be represented as the phenotype E: epibranchial 4 bone [in_taxon X], Q: width^increased_in_magnitude_relative_to(E:epibranchial 4 bone in_taxon Y) asserted for twenty-five *Cyprinella* species or narrow (in two other species) and the phenotype E: epibranchial 4 bone [in taxon Y], Q: width^decreased_in_magnitude_relative_to[E:epibranchial 4 bone in_taxon X] asserted for *Cyprinella xaenura* and *Cyprinella pyrrhomelas*. Note that PATO includes some pre-coordinated relative qualities such as *decreased size*, which means decrease in size when compared to comparable members of the same species – the use of this in inappropriate here.

**Literature Cited**
